# Adsorption of ice-binding proteins onto whole ice crystal surfaces does not necessarily confer a high thermal hysteresis activity

**DOI:** 10.1038/s41598-022-19803-3

**Published:** 2022-09-14

**Authors:** Tatsuya Arai, Akari Yamauchi, Yue Yang, Shiv Mohan Singh, Yuji C. Sasaki, Sakae Tsuda

**Affiliations:** 1 Department of Advanced Materials Science, Graduate School of Frontier Sciences, The University of Tokyo, 7H8 #609 Kiban Bldg., 5-1-5 Kashiwanoha, Kashiwa, Chiba 277-8561 Japan; 2 AIST-UTokyo Advanced Operando Measurement Technology Open Innovation Laboratory, National Institute of Advanced Industrial Science and Technology (AIST), Kashiwa, 277-0882 Japan; 3 Hibernation Metabolism, Physiology and Development Group, Institute of Low Temperature Science, Hokkaido University, Sapporo, 060-0819 Japan; 4 Department of Botany, Banaras Hindu University (BHU), Varanasi, 221005 India

**Keywords:** Biogeochemistry, Proteins

## Abstract

Many psychrophilic microorganisms synthesize ice-binding proteins (IBPs) to survive the cold. The functions of IBPs are evaluated by the effect of the proteins on the nonequilibrium water freezing-point depression, which is called “thermal hysteresis (TH)”, and the inhibitory effect of the proteins on the growth of larger ice crystals, which is called “ice recrystallization inhibition (IRI)”. To obtain mechanical insight into the two activities, we developed a modified method of ice affinity purification and extracted two new IBP isoforms from *Psychromyces glacialis*, an Arctic glacier fungus. One isoform was found to be an approximately 25 kDa protein (PsgIBP_S), while the other is a 28 kDa larger protein (PsgIBP_L) that forms an intermolecular dimer. Their TH activities were less than 1 °C at millimolar concentrations, implying that both isoforms are moderately active but not hyperactive IBP species. It further appeared that both isoforms exhibit high IRI activity even at submicromolar concentrations. Furthermore, the isoforms can bind to the whole surface of a hemispherical single ice crystal, although such ice-binding was generally observed for hyperactive IBP species. These results suggest that the binding ability of IBPs to whole ice crystal surfaces is deficient for hyperactivity but is crucial for significant IRI activity.

## Introduction

Glaciers cover 10% of the land surface on Earth and are considered to provide a habitat for various living organisms^1,2^. Among these creatures, a vast number of microorganisms in glaciers are known to develop survival strategies to adapt to ice-laden environments, and these strategies include adaptation to low availabilities of water and nutrients and preventing ice-induced damage by producing cryoprotective substances. These substances include cations, phosphates, nucleotides, amino acids, polyols (ex. glycerol), sugars (ex. trehalose), and proteins (ex. ice-binding protein). The cryoprotective substances are now utilized in industrial and medical fields, and how each substance functions to control ice-crystal growth must be clarified^3^ to develop further advancements in cryo-technology.

Ice-binding protein (IBP) is the most promising cryoprotective substance and uniquely binds to embryonic single ice crystals in a supercooled aqueous solution^4^. Notably, IBP is sometimes called antifreeze protein (AFP). The IBP-adsorbed ice crystals cannot undergo further growth. IBPs have been identified from various kinds of cold-adapted organisms, such as fish^5^, insects^6^, plants^7^, and microorganisms^8^. Microorganisms synthesize a β-helical IBP that lacks the structural regularity due to the nonrepetitive nature of the amino acid sequence. Recent studies have demonstrated that many IBP molecules locate some ice-like organized waters on their flat surface^9,10^, which may undergo complementary binding to the waters that comprise the basal, prism, and/or pyramidal planes defined for an embryo single ice crystal. Notably, the unit structure of a single ice crystal at an atomic scale is a hexagonal cylinder, which is defined by three equivalent *a*-axes (*a*_1_–*a*_3_) that are perpendicular to the *c*-axis. The basal planes are the two top planes in the hexagonal unit. The *c*-axis penetrates the center portion of the basal planes of the hexagonal cylinder.

The ability of microorganism-derived β-helical IBPs to bind ice can be evaluated by two physical quantities, including thermal hysteresis (TH) and ice recrystallization inhibition (IRI). The TH activity is an IBP-induced depression of the nonequilibrium freezing point (*T*_f_) of water and does not cause a significant change in the melting point (*T*_m_). The TH value is hence evaluated as a difference between these two points for an IBP solution (i.e., TH = *T*_m_−*T*_f_)^11^. The known IBPs have been categorized into moderately active and hyperactive groups based on the maximal TH values evaluated for each solution^12^. Most insect-derived IBPs belong to the hyperactive group, which exhibits 2 − 6 °C of high TH values when evaluated at only micromolar concentrations. In contrast, ordinary fish-derived IBPs are categorized into the moderately active group, which exhibits less than 1.5 °C at millimolar concentrations. In the frozen state of water, larger ice crystals grow at the expense of smaller ones, and this process is called ice-recrystallization. When an IBP exerts strong IRI activity, the IBP maintains an ice crystal size and minimizes freezing damage to the host organism. The fluorescence-based ice plane affinity (FIPA) pattern provides further information about the target ice planes of IBP species^13^. By submerging a single ice crystal hemisphere of 2–3 cm in diameter into a vessel that contains a solution of fluorescence-labeled IBPs, their adsorptions show specific patterns on the ice hemisphere under UV light. When six equally distant ellipses are illuminated on the equator, it indicates that the IBP binds to six equivalent prism planes of a single ice crystal. Similarly, the illumination of six ellipses on the mid-latitude implies that the IBP is binding to six equivalent pyramidal planes. Entire illumination of the hemisphere implies that the IBP sample binds to the whole ice crystal plane.

It has been shown that the TH values of microbial IBPs are distributed over a wide range (0.1 − 4.0 °C)^14,15^. The IRI activity of microbial IBPs tends to be high compared to that of insect- and fish-derived IBPs^16^. The FIPA patterns of microbial IBPs exhibit variations. Since it was demonstrated that hyperactive IBPs from insects show the entire illumination of the FIPA analysis, the binding ability to the whole crystal surface, including the prism, pyramidal, and basal plane of a single ice crystal, was regarded as a key determinant for the high TH activity. The present study examined this hypothesis through by characterizing new microbial IBP samples, for which we carefully examined both IRI and TH activities as well as the FIPA pattern.

IBP from a cold-adapted dimorphic fungus, *Psychromyces glacialis*, was previously identified^17^. This microorganism was previously called *Rhodotorula svalbardensis*, but it was recently changed to the above name to be identified as a member of *Glaciozyma* species^18^. *P. glacialis* has been isolated from a variety of glacial environments, such as cryoconite holes, dark ice, snow, subglaciers, and glacier meltwater in Svalbard, Norway^19^ and Greenland^20^. The microorganism was not found in the other near-polar regions, suggesting that *P. glacialis* is highly endemic to specific areas. A preliminary study showed that the IBP from *P. glacialis* (PsgIBP) could modify the shape of a single ice crystal, which suggested that IBP production is beneficial for the survival of this fungus in icy environments. Here, we re-evaluated the biochemical properties and ice-binding ability of PsgIBP. We found that the PsgIBP sample is a mixture of two isoforms, a relatively small isoform (PsgIBP_S) and a dimer-forming large isoform (PsgIBP_L), for which moderate TH and significant IRI activities were detected. The FIPA analysis further showed its ability to bind to the whole plane of a single ice crystal hemisphere. The correlation between TH, IRI, and FIPA and its physiological role will be discussed in terms of the cold-survival mechanism of this microorganism.

## Results

### Psychromyces glacialis secretes a mixture of two PsgIBP isoforms

**Figure 1:**
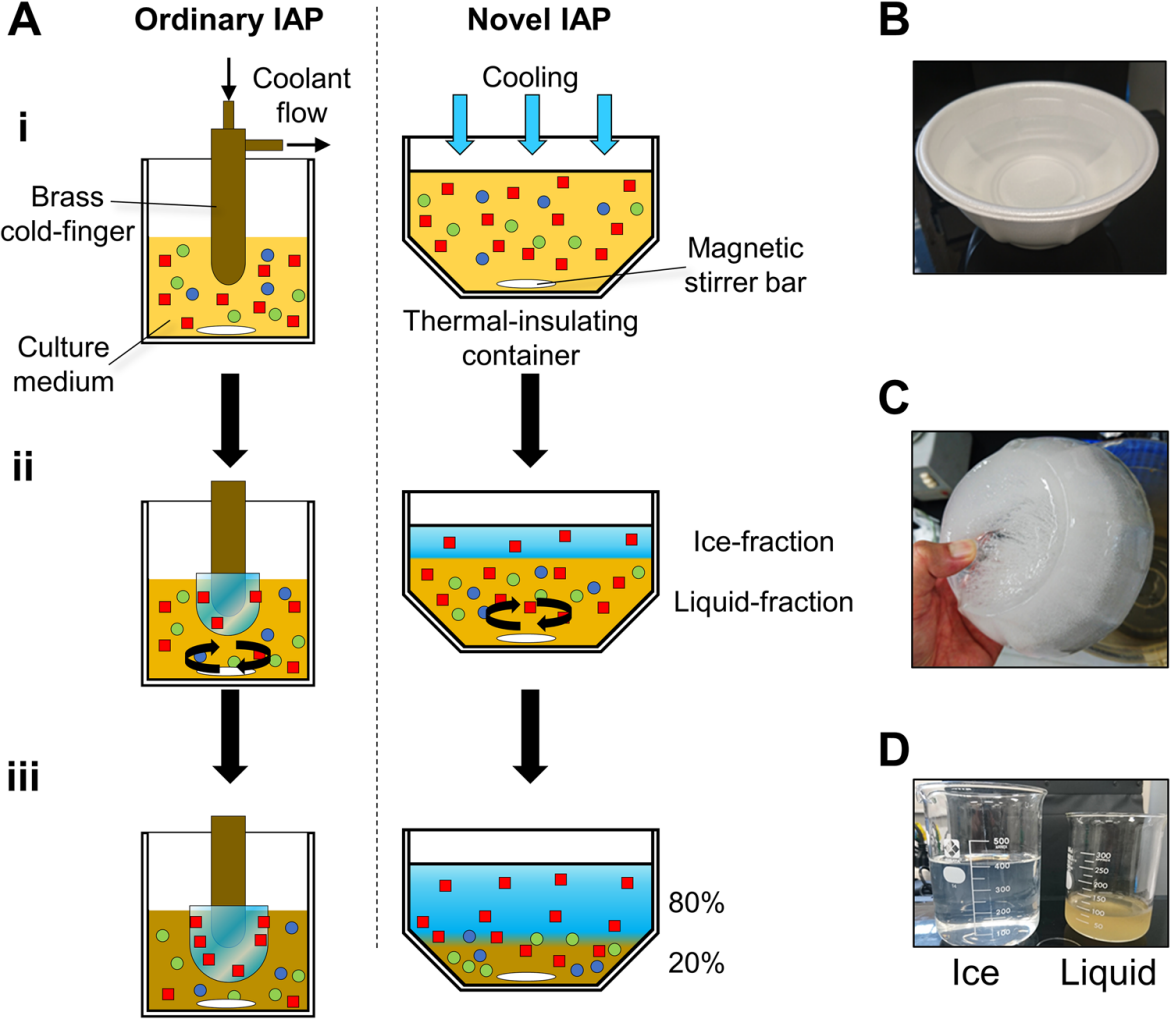
A modified ice-affinity purification method to obtain PsgIBP samples. (**A**) Comparison between ordinary and novel IAPs. In the ordinary IAP, a brass cold-finger with a flow of cold ethylene glycol was soaked in sample solution (i) and was then surrounded by the frozen sample containing IBP (ii, iii). In the novel IAP method, a 500 mL solution containing IBP was placed into a thermal insulation vessel in a − 3 °C-refrigerator. The − 3 °C incubation for 10–12 h induced one-directional freezing from the top to the bottom of the solution, which led to the generation of an ice fraction. To be concentrated in the liquid fraction, the ice fraction only adsorbed IBPs (red squares) and excluded contaminants (blue and green spheres). (**B**) The polystyrene foam thermal insulation vessel (600 mL). (**C**) The ice fraction containing the IBPs removed from the vessel. (**D**) Comparison of the ice- and liquid fractions after thawing.

**Figure 2:**
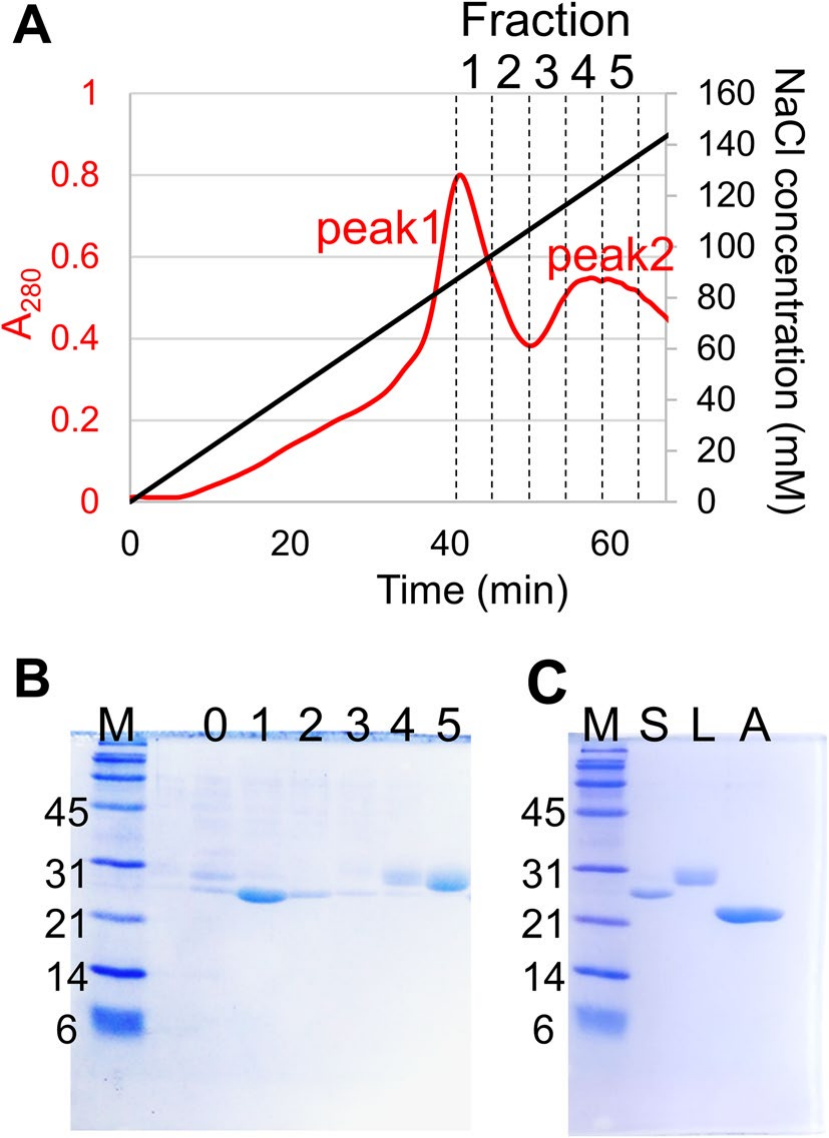
Purification of PsgIBP. (**A**) An elution profile of anion-exchange chromatography for the recombination extracts containing PsgIBP. (**B**) SDS–PAGE electrophoretograms of fractions 1–5 obtained by anion-exchange chromatography. Lane M, molecular weight marker; 0, before chromatography; 1–5, the fractions collected in (**A**). (**C**) SDS–PAGE electrophoretograms of purified PsgIBP_S and PsgIBP_L. Lane S, PsgIBP_S; Lane L, PsgIBP_L; Lane A, recombinant AnpIBP (22 kDa) for comparison. The samples were stained with Coomassie brilliant blue R-250. The original gels are presented in Supplementary Fig. 1A and 1B.

*Psychromyces glacialis* cells were originally isolated from a cryoconite hole in Svalbard and were cultivated in our laboratory. Since a preliminary study showed that *P. glacialis* secretes IBPs into the extracellular space^17^, we purified native PsgIBP from the culture supernatant. Ice-affinity purification (IAP), a uniquely developed method to purify IBPs^21^, was utilized for the initial step of our purification procedure. In the ordinary method, IBP is obtained from the ice phase as the IBP undergoes irreversible adsorption to the growth-induced ice fronts, while the other molecules cannot be incorporated into the ice phase. A merit of the IAP method is that the method can always be used unless the molecular weight (MW), isoelectric point, and/or thermostability of the IBP is unknown. The ordinary IAP method uses a frosty copper probe, which necessitates special instrumental settings (Fig. 1A, left), so we developed a modified IAP method that simplifies the purification process and increases the volume of the sample. The right side of Fig. 1A shows a schematic diagram of the modified IAP method. As shown, a polystyrene foam thermal insulating vessel (600 ml) that contains the IBP culture supernatant was placed in an incubator set to − 3 °C (Fig. 1B) to induce one-directional freezing from the top to the bottom. After a thin ice layer formed on the top surface of the supernatant, stirring was initiated to prevent the accumulation of the contaminants at the ice-water interface. The ice phase was further grown at − 2 °C for 10–16 h until 80% of the sample was frozen. The ice fraction that contained IBP was then removed from the vessel, rinsed with cold water, and melted at room temperature (Fig. 1C). Figure 1D shows a comparison of the melted ice fraction and the remaining liquid fraction. The materials with a brown color that were depicted in the liquid fraction, which are difficult to remove by the other methods, were clearly excluded from the ice fraction. The ice fraction was subjected to anion-exchange chromatography, and its elution profile is shown in Fig. 2A. The chromatogram had two main peaks, both of which showed antifreeze activity. The SDS–PAGE performed for the ice fraction shows that it consists of two major polypeptides, in which the MWs were approximately 25 and 28 kDa, respectively (Fig. 2B, Lane 0). The polypeptides are mostly identical to the size of the other microbial IBPs. These two protein bands showed almost the same intensity on SDS–PAGE with CBB staining, indicating that their expression levels should be almost the same. The SDS–PAGE for the two peaks in the chromatogram (Fig. 2A, fractions 1–5) indicated that two proteins were successfully separated (Fig. 2B, Lanes 1–5). The two peaks were separately collected and further purified by size-exclusion chromatography (SEC) by employing a Superdex200 column. Each protein was detected as a single band by SDS–PAGE (Fig. 2C). We assigned the two polypeptides to IBP and named them PsgIBP_S and PsgIBP_L for the smaller (25 kDa) and larger (28 kDa) isoforms, respectively.

### PsgIBP_S is a monomer, while PsgIBP_L forms a dimer

**Figure 3:**
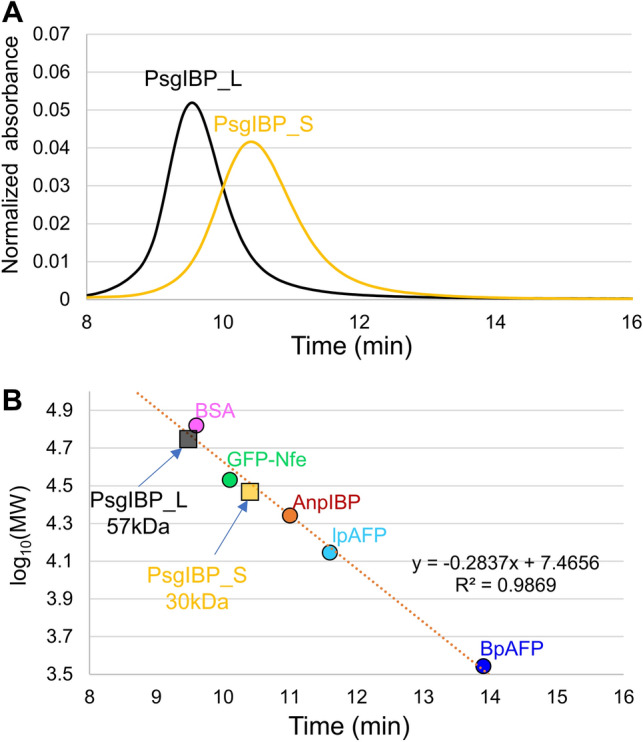
Molecular weight determination for the PsgIBP isoforms based on analytical size exclusion chromatography (SEC). (**A**) The elution profiles of PsgIBP_S, PsgIBP_L, and reference proteins obtained by using Superose12 gel chromatography. (**B**) Molecular weight (log_10_Mw) dependence on SEC elution time. BSA, bovine serum albumin (66 kDa); GFP-Nfe, green-fluorescence protein-labeled notched fin eelpout-derived IBP (34 kDa); AnpIBP, *Antarctomyces psychrotrophicus*-derived IBP (22 kDa); lpAFP, Longsnout poacher-derived IBP (14 kDa); BpAFP, Barfin plaice-derived IBP (3.5 kDa). On the basis of the calibration line, the MW of approximately 57 and 30 kDa were evaluated for PsgIBP_L and PsgIBP_S, respectively.

In the chromatography process with the Superdex200 column, the elution time of PsgIBP_L was much faster than that of PsgIBP_S despite their very similar MWs verified by SDS–PAGE. Since an IBP from the Arctic yeast *Glaciozyma* sp. A30Y (LeIBP) forms a dimer^14^, we speculate that PsgIBP_L undergoes dimerization similar to LeIBP. To estimate the MW of the two purified isoforms of PsgIBP, SEC was performed using a Superose12 column instead of a Superdex200 column. Figures 3A and S2 show the elution profiles of the PsgIBP isoforms and those with five reference proteins, respectively. As shown, each isoform was eluted as a single peak, and significant differences were observed in the times needed to elute each isoform. The elution time of PsgIBP_S lies between that of the GFP-nfeAFP fusion protein (34 kDa) and AnpIBP (22 kDa). Hence, the MW of this isoform was estimated to be 30 kDa (Fig. 3B), which is almost the same as that on SDS–PAGE (25 kDa). On the other hand, the elution time of PsgIBP_L was much faster than that of the GFP-nfeAFP fusion protein and was almost the same as that of BSA (66 kDa). The MW of PsgIBP_L was therefore evaluated at 57 kDa, which is approximately 2 times larger than that determined by SDS–PAGE. These results suggest that PsgIBP_L forms a dimer in solution. Note that the detection of PsgIBP_L and BSA at almost the same elution time is due to the resolution limit of this method; the error increases for the larger or smaller proteins. However, the high R2 value (0.9869) shown in Fig. 3B supports the appropriateness of the analysis. It should be noted that this dimerization is not attributed to disulfide bonds, since the MW position of PsgIBP_L in SDS–PAGE (28 kDa) was not changed regardless of the presence or absence of the reductant dithiothreitol (data not shown).

### Both PsgIBP isoforms exhibit moderate thermal hysteresis activity

**Figure 4:**
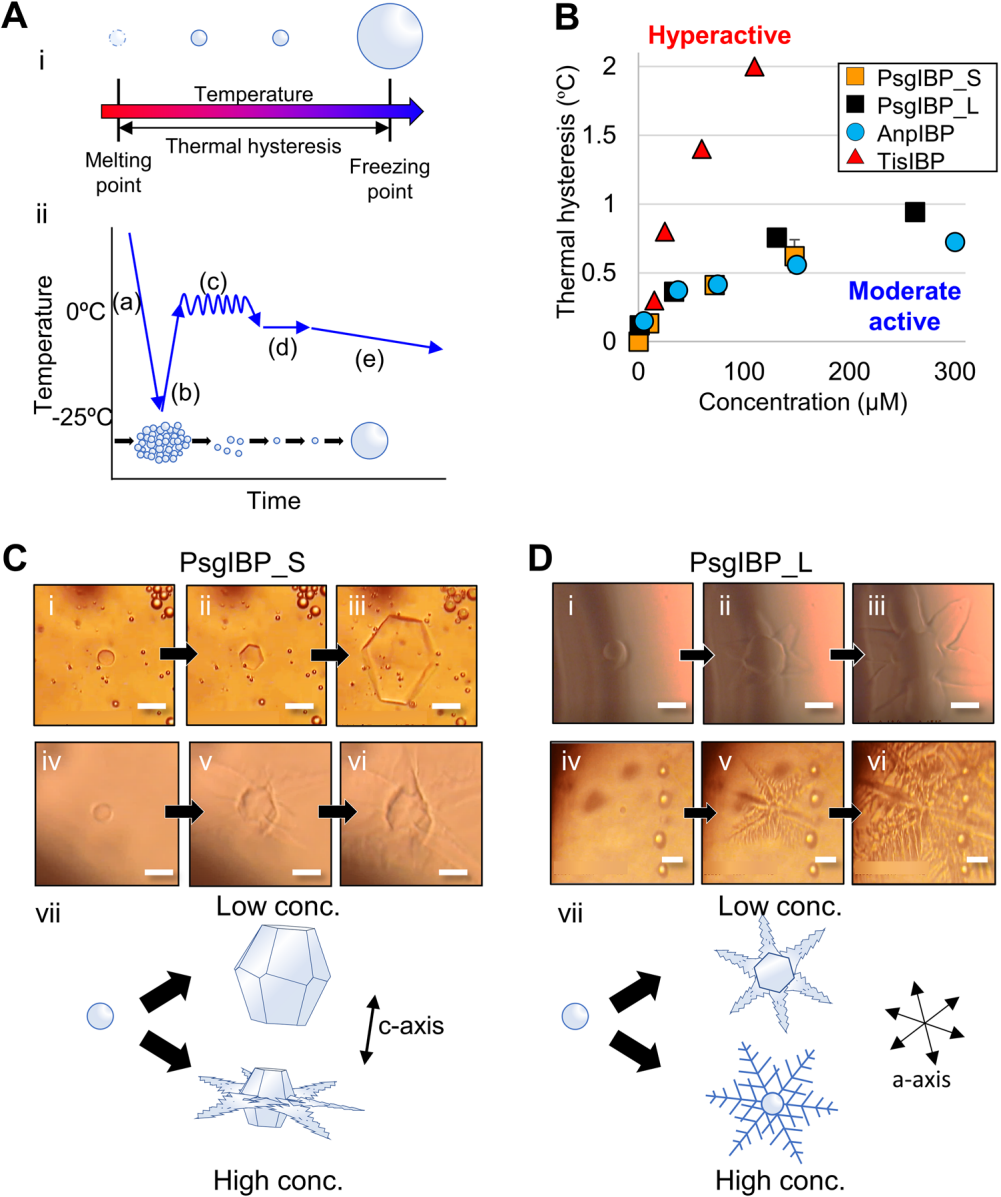
Thermal hysteresis (TH) and ice-shaping activities of PsgIBP isoforms. (**A**-i) TH is measured as a temperature range called the “TH gap” between the melting (*T*_m_) and freezing points (*T*_f_) of a single ice crystal (white ball) that was prepared in the sample solution. The crystal neither grew nor melted within the TH gap, while it exhibited bursting growth at *T*_f_. (**A**-ii) The solution was flash frozen (a) to form a polycrystalline state of single ice crystals (b). The solution was then warmed up to 0 °C to nullify most of the crystals and was repeatedly cooled and warmed to secure a single ice crystal in sample (d). The sample was then cooled at a rate of 0.1 °C/min to measure the *T*_*f*_ of the single ice crystal. (**B**) Concentration dependence of TH of PsgIBPs. The dependence of TisIBP (hyperactive IBP) and AnpIBP (moderately active IBP) was also depicted as the reference. The error bars show the standard deviation based on n = 3 replicates. (**C** and **D**) The morphological change of a single ice crystal observed for PsgIBP_S and PsgIBP_L at low (i–iii) and high (iv–vi) concentrations. Scale bar = 25 μm. The data show a single ice crystal within the TH gap (i and iv) and its bursting growth below *T*_*f*_ (ii, iii and v, vi). (vii) Schematic interpretations of the observed changes in the ice crystal.

It has been shown that the TH activities of microbial IBPs are variable (0.1–4.0 °C) despite their structural similarity^8^. Here, we measured the TH activity for the purified PsgIBP_S and _L samples by using a homemade photomicroscopic system, in which the stage temperature was manipulated to monitor the crystal growth of a single ice crystal that was generated in the sample solution (see Fig. 4Ai and the “Materials and methods” section). The TH value, a parameter that shows the inhibition of IBP ice growth, was calculated as the difference between the *T*_m_ and *T*_f_ of the single ice crystal (Fig. 4Aii). Figure 4B shows the TH activities evaluated for the PsgIBP isoforms and compares the data with those evaluated for the known microbial IBPs^22,23^. As shown, the TH value of PsgIBP_S was 0.63 °C at 150 μM and that of PsgIBP_L was 0.76 °C at 130 μM. Several IBPs have been reported to increase TH activity when the two isoforms are mixed^24,25^. To examine whether this is true in the case of PsgIBP, we mixed purified PsgIBP_L and PsgIBP_H at a ratio of 1:1 and measured the TH activity. The TH activity of the mixed PsgIBPs was 0.79 ± 0.05 °C at 130 μM, which was not significantly higher than that of a single isoform. The PsgIBP isoforms may not work cooperatively in terms of TH activity.

It should be noted that the TH activity measurement for PsgIBP isoforms at higher concentrations was extremely difficult to obtain because the size of an ice crystal at higher PsgIBP concentrations becomes very small, which makes it difficult to leave one single ice crystal in the sample solution. Although the TH activity of the PsgIBP_L isoform is slightly higher than that of the PsgIBP_S isoform, the two activities are comparable to that of a previously reported moderately active *Antarctomyces psychrotrophicus* IBP (AnpIBP) and much lower than that of the hyperactive *Typhula ishikariensis* IBP isoform 8 (TisIBP8). On the basis of the TH activity measurements, it could be said that both PsgIBP isoforms are categorized into the moderately active group of IBPs.

It is well known that the binding of IBPs within the temperature range between *T*_m_ and *T*_f,_, which is called the “TH gap”, modifies the shape of a single ice crystal into a hexagonal plate, a hexagonal bipyramid, a hexagonal trapezohedron, or a lemon-like morphology. Notably, the two PsgIBP isoforms affected the shape of a single ice crystal differently (Fig. 4C and D), although they exhibited similar TH activity. Within the TH gap, PsgIBP_S stopped growing a single ice crystal without changing the ice shape, leading to the formation of a small, rounded ice crystal (Fig. 4Ci). At the lowest limit of TH, this ice crystal, on which the PsgIBP_S isoform adsorbed, suddenly started growing toward both the *a*- and *c*-axes and changed into a bipyramidal shape (Fig. 4C ii and iii) at the lower protein concentrations. This bipyramidal ice crystal is similar to that typically observed in the moderately active IBPs that bind to prism and/or pyramidal planes^12^. The only difference between PsgIBP_S and the moderately active IBPs is that the two tips of the PsgIBP_S-bound ice bipyramid are truncated (Fig. 4Ciii). On the other hand, when the concentration of PsgIBP_S is higher, the rounded single ice crystal bursts toward both the *a*- and *c*-axes (Fig. 4Civ–vi). This concentration-dependence of an IBP’s ability to shape ice was similarly observed for TisIBP isoform 7^26^, which can bind to the whole ice crystal plane, although its TH activity lies between the hyper and moderate activities.

Similar to PsgIBP_S, the PsgIBP_L isoform can stop the growth of a single ice crystal without changing the rounded ice crystal shape within the TH gap (Fig. 4Di and iv). However, the bursting pattern of the ice crystal was different from that observed for PsgIBP_S. Namely, the ice crystal always grew in six *a*-axis directions regardless of the IBP concentration. The crystal exhibited a flower-like bursting growth at 1 μM PsgIBP_L (Fig. 4Di–iii) and a vein-like pattern at 130 μM PsgIBP_L (Fig. 4Div–vi). This bursting ice crystal growth was typically observed for the hyperactive IBP species that bound to the whole ice crystal surface, including the prism, pyramidal, and basal planes^23,27,28^.

### Both PsgIBP isoforms bind to the whole ice plane despite their moderate TH activities

**Figure 5:**
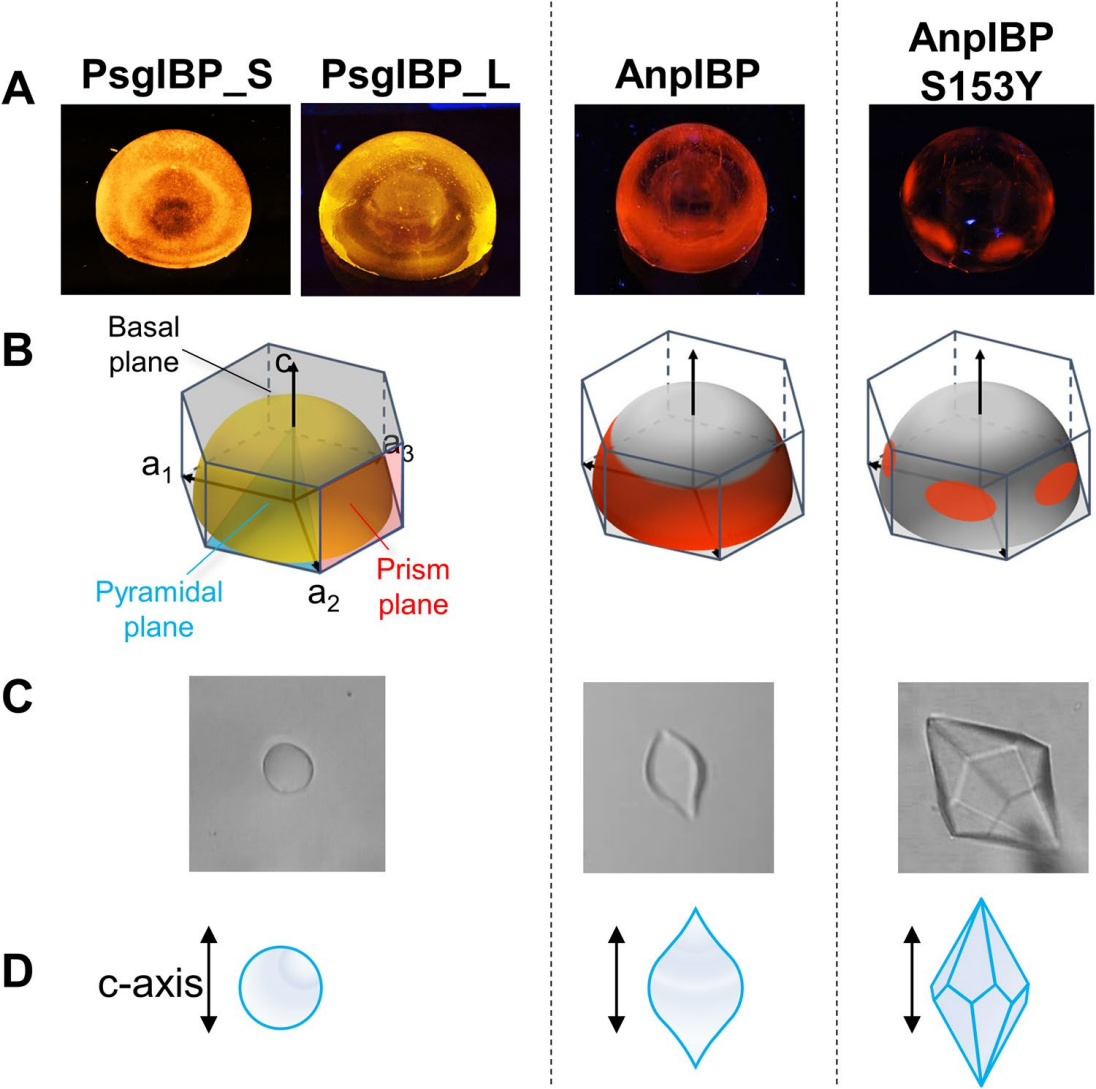
Fluorescence-based ice plane affinity (FIPA) patterns and the ice crystal morphologies observed for PsgIBPs. (**A**) The patterns of PsgIBP_S, PsgIBP_L, AnpIBP, and AnpIBP_S153Y visualized on a single ice crystal hemisphere. (**B**) Location of the hexagonal ice crystal planes on the ice hemisphere. The polar, equatorial, and midlatitude regions correspond to the basal, prism, and pyramidal planes, respectively. (**C**) Photomicroscope images of the embryo single ice crystals observed in the TH gap. (**D**) Illustrative interpretations of the images.

Although both PsgIBP isoforms exhibited moderate TH activity, they could modify the single ice crystal into a rounded globular shape, which was observed for the IBPs that exhibited hyper TH activity. To examine the ice-binding properties of PsgIBPs and their relevance to TH activity in more detail, we observed the FIPA pattern on the single ice crystal hemisphere. In this method, a single ice-crystal hemisphere with a diameter of 3– 4 cm was immersed into a − 8 °C-chilled solution that contained a fluorescence-labeled IBP. Following immersion for 2–3 h, the crystal was removed from the solution and was observed under UV light, which visualizes the fluorescent IBP adsorbed on the single ice crystal hemisphere and clarifies the IBP-bound ice crystal planes. Figure 5A shows the UV-illuminated hemispheres that indicate the FIPA pattern of the PsgIBP_L and PsgIBP_S labeled with the fluorescence reagent 5(6)-TAMRA-X SE. For comparison, the figure shows the FIPA patterns observed for the other microbial IBPs, AnpIBP and the S153Y mutant of AnpIBP. In an ice crystal hemisphere, a top hexagonal plane (gray), six side planes (orange), and six inclined planes (green) correspond to the basal, prism, and pyramidal planes of a single ice crystal, respectively (Fig. 5B, left). Note that the ice crystal hemisphere is entirely illuminated when IBP binds to all crystal planes. Such an entire illumination was unexpectedly observed for both the PsgIBP_L and PsgIBP_S isoforms, as shown in Fig. 5A. The ice-binding ability to the whole ice crystal plane is consistent with the photomicroscopic observations for the PsgIBP_L and PsgIBP_S isoforms (Figs. 4C and 5C). Namely, no facetted morphology but rather a rounded single ice crystal was generated in a solution containing the PsgIBP isoforms, which was attributable to their ability to bind the whole ice crystal planes. It is surprising that moderately active IBPs bind to the whole ice plane because such ice binding has been observed for only hyperactive IBPs^23,27,28^.

By comparing the FIPA pattern in Fig. 5A, the ability of the moderately active PsgIBP isoforms to perform whole ice-plane binding was verified. AnpIBP and its S153Y mutant are also known as moderately active IBPs. The AnpIBP binds to the plural prism planes and constructs the equator region of the ice hemisphere, which is called the “prism ring” (Fig. 5B)^10^. AnpIBP cannot bind to the basal plane, which results in orbital illumination of the ice hemisphere. This ice-binding ability of AnpIBP modifies a single ice crystal into a lemon-like morphology (Fig. 5C) that is characterized by two sharp tips along the *c*-axis. In the case of the AnpIBP_S153Y mutant, the crystal only adsorbs onto pyramidal planes, which gives six distinct patches on the mid-latitude of the single ice crystal hemisphere. The AnpIBP_S153Y mutant modifies a single ice crystal into a hexagonal bipyramid (Fig. 5D, right end). As shown, the PsgIBP isoforms shape a single ice crystal into a rounded shape (Fig. 5C and D, left), which is similar but not identical to the lemon-like shape and totally different from the hexagonal bipyramid shape. These results support that the PsgIBP isoforms have a unique manner of binding ice, as the isoforms bind to the whole ice crystal plane, although they are categorized into moderately active IBP species on the basis of their TH activity.

### PsgIBPs exhibit strong IRI activity even at nanomolar concentrations

**Figure 6:**
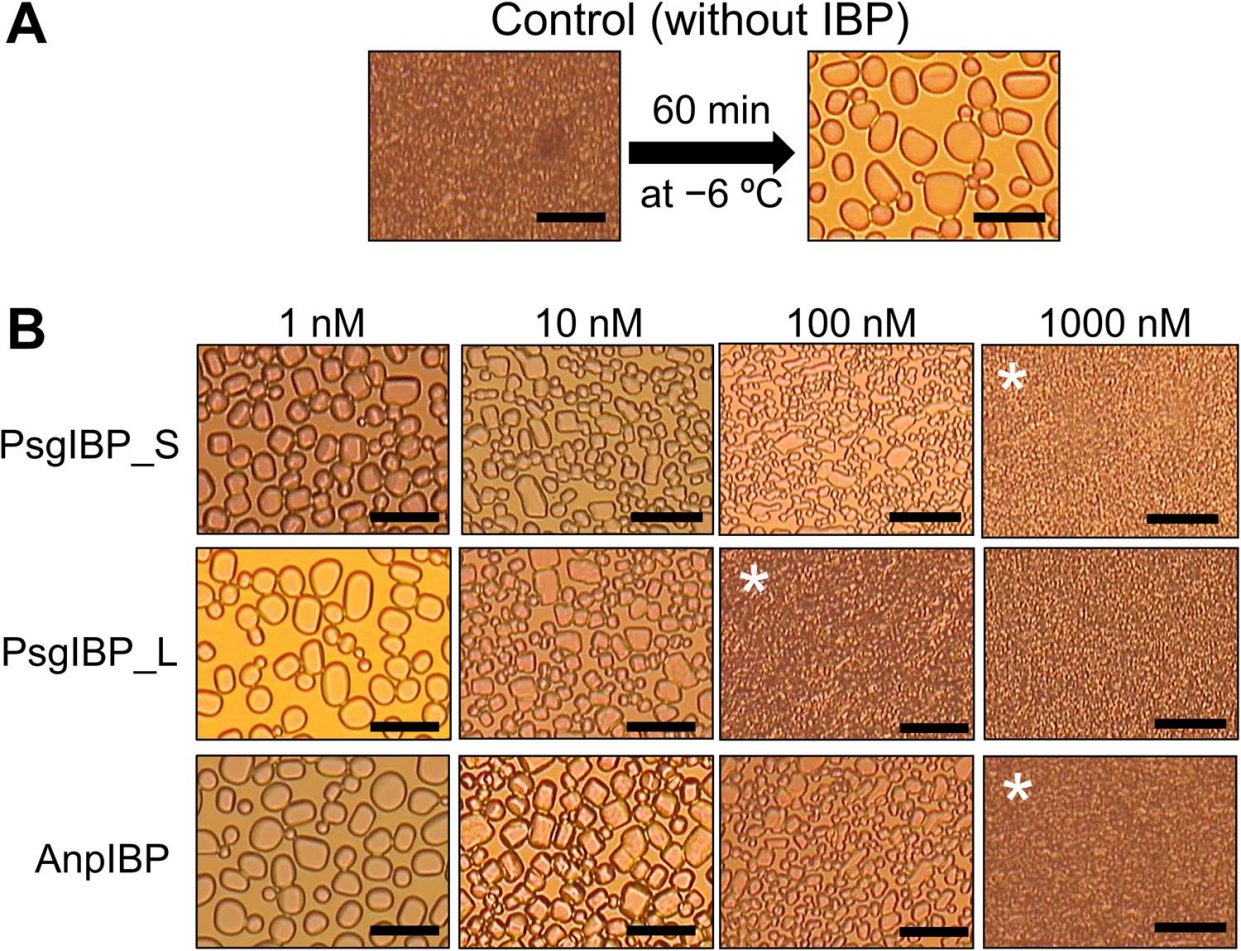
Concentration dependence of the ice recrystallization inhibition (IRI) activity of PsgIBPs. (**A**) Photomicroscope images to observe an ordinary ice recrystallization (IR) process of frozen water containing 30% sucrose. Left: Observation of the numerous tiny single ice crystals generated in frozen water. Right: Change in the ice crystals owing to IR after 60 min of incubation at − 6 °C. (**B**) Photomicroscope images to observe the IRI activity of PsgIBPs in a series of ten-fold serial dilutions. The asterisks indicate the minimum concentration necessary for IRI. Scale bar = 50 µm.

The ice recrystallization inhibition (IRI) activity of the microbial IBPs tends to be high compared with that of fish and insect IBPs, and it was speculated that ice recrystallization is among the main causes of damage to microorganisms due to freezing^16^. We measured the IRI activity of the PsgIBP_S and _L isoforms by observing a time-dependent change in the ice crystals in their solutions that contained 30% sucrose. We prepared 1, 10, 100, and 1000 nM solutions of each isoform, which were placed in an incubator that was set to − 6 °C, and their photomicroscope images were compared after 60 min. As a reference, Fig. 6A shows the images of the frozen solution without IBP before and after incubation at − 6 °C for 60 min. Before the incubation, numerous tiny single ice crystals were observed in the frozen solution, in which the outlines of the ice crystals could not be observed with the resolution of the microscope because the ice crystals were too small. These ice crystals underwent recrystallization and became larger during the 60-min incubation, which concomitantly resulted in a dramatic decrease in the ice crystal number (Fig. 6A). In contrast, it appeared that the addition of only 1 − 10 nM PsgIBP_L or PsgIBP_S samples inhibited ice recrystallization and effectively decreased each ice crystal size (Fig. 6B). When comparing the images of PsgIBP_S or PsgIBP_L at 100 nM concentration, the image of PsgIBP_L contained more tiny single ice crystals and is identical to the control image before incubation. The single ice crystals become tiny when 1000 nM PsgIBP_S was added, implying that compared to PsgIBP_L, more PsgIBP_S is needed for complete IRI. On the basis of these results, the critical concentration for IRI activity was evaluated at 100 nM and 1000 nM for PsgIBP_S and PsgIBP_L, respectively. It has been reported that AnpIBP possesses effective IRI activity^22^, and we further examined its activity for comparison. As shown in the lower panels of Fig. 6B, AnpIBP exhibited no significant IRI activity at 1 nM and required 1000 nM for complete IRI. The image of 100 nM AnpIBP is similar to that of 100 nM PsgIBP_S. These results suggest that the IRI activities of AnpIBP and PsgIBP_S are almost the same, and the activity of PsgIBP_L is higher than that of the two IBP isoforms.

## Discussion

IBPs have been categorized into hyperactive and moderately active groups based on their TH activities. Many researchers have investigated what kind of factor determines the strength of TH activity and how it correlates with IRI activity. In the case of insect IBPs, which typically show 5 °C or higher TH activity, basal-plane affinity is thought to be the key determinant for hyperactivity^12^ because most of them bind to the basal plane in addition to the prism and pyramidal planes. In the case of microbial IBPs, a species binding to the basal plane does not always exhibit such a significant TH activity. For example, arctic yeast LeIBP binds to the basal and 1st prism planes but exhibits a TH activity of only 0.9 °C at millimolar concentrations^14^. An IBP derived from perennial ryegrass *Lolium perenne* is another example that exhibits affinity to the basal plane with weak TH activity^29^. These observations suggested that the ability of basal-plane binding is not sufficient for hyperactivity. The current study introduces two new IBP species that possess moderate TH activity and whole ice-plane affinity.

The binding of PsgIBP_S inhibits the growth of a single ice crystal and causes the crystal to form a rounded shape within the TH gap (Fig. 4Ci). The ability to hold a tiny, rounded ice crystal has often been observed for hyperactive IBPs that bind to the whole ice crystal plane. When the temperature decreases exceeded the *T*_f_, the PsgIBP_S-bound ice crystal was changed into a truncated hexagonal bipyramid at lower concentrations, which is typically observed for moderately active IBPs that generally do not bind to the basal plane^12^. When the PsgIBP_S concentration was higher, the bursting ice growth progressed toward both the *a*- and *c*-axis directions, which was similarly observed for hyperactive IBPs. That is, PsgIBP_S exhibits signs of both hyper and moderate activities. Based on these results, we speculated that although PsgIBP_S can bind multiple ice surfaces, including the basal planes, the binding affinity to the basal plane is weak compared with that of ordinary hyperactive IBPs. An IBP from a bacterial symbiont of a psychrophilic ciliate *Euplotes focardii* was reported to bind weakly to most of the pyramidal planes, while it can bind strongly to limited portions near the basal planes and near the secondary pyramidal planes^30^. This mechanism uniquely causes a “Saturn-shaped ice burst pattern” below the freezing point. In the case of PsgIBPs, such strong and weak binding was not observed in the FIPA analysis (Fig. 5). Thus, a developed method would be necessary to determine the ice binding strength of IBPs to specific ice planes.

In the case of PsgIBP_L, the ice crystal burst occurred in six *a*-axis directions, exhibiting a vein-like ice growth pattern even at lower concentrations, which was similarly observed for the other hyperactive IBPs^23,27,28^. Unlike PsgIBP_S, PsgIBP_L always exhibited an ice crystal burst toward six *a*-axes even at a very low concentration (1 µM), suggesting that this IBP has a strong basal-plane affinity. Why does PsgIBP_L exhibit moderate TH activity? We speculate that dimerization might help answer this question. The dimerization was identified for the LeIBP from the Arctic yeast *Glaciozyma* sp. AY30^31^. In the X-ray crystal structure of the LeIBP dimer, two IBP molecules form a complex in a back-to-back manner through their C-terminal long tails to expose their ice-binding sites at opposite sides. When the C-terminal tail of LeIBP was removed, its dimer formation was destroyed, and its TH activity was increased compared with that of the wild type. Notebly, the present *P. glacialis* is phylogenetically close to *Glaciozyma* sp. AY30^18^. On the basis of these results, it is speculated that in PsgIBP_L, a loss of freely accessible ice-binding sites due to dimerization leads to lower TH activity in a similar manner to that of LeIBP.

The present study demonstrated that both PsgIBP isoforms bind entirely to ice crystal planes (Fig. 5) and effectively inhibit ice recrystallization (Fig. 6). The IBP binding to multiple ice planes was assumed to contribute to the IRI activity^32^, since the IBP binding to multiple ice planes will keep the water molecules away in both water–adsorption and water-desorption processes at the ice-water interface. Thus, it may be assumed that the whole-plane binding of PsgIBPs is crucial for their high IRI activity, unless its basal-plane affinity is weak. Gruneberg et al. recently examined a correlation between TH and the IRI of fifteen diverse IBPs and pointed out that the IRI activity increases proportionately to their molecular weight^16^. This assumption holds true in our experiment (Fig. 6B), as the dimer-forming isoform PsgIBP_L exhibited higher IRI activity than that of the monomer isoforms PsgIBP_S or AnpIBP.

*Psychromyces glacialis* was initially isolated from a cryoconite hole, which is a water-filled hole on the glacier surface, and has also been isolated from various glacier environments in Svalbard and Greenland. In these niche environments, microorganisms are exposed to freeze–thaw cycles that occur frequently. For example, the temperature of cryoconite holes in Svalbard ranges from 0.2 to 1.9 °C during summer^17^, while these holes are frozen in the winter season^33^. Therefore, *P. glacialis* must withstand repetitive freezing and thawing cycles and should be equipped with strategies to minimize damage due to freezing. Fujiu et al. recently proposed that the Antarctic yeast *Glaciozyma antarctica* synthesizes IBPs and extracellular polysaccharides to retain water around the cells^34^. PsgIBP may also play this physiological role to prevent the extracellular space around the host cells from freezing. The whole plane binding of PsgIBPs stops ice crystal growth efficiently to secure the unfrozen space around the host cells. Fujiu et al. also showed that *G. antarctica* can grow on a frozen potato dextrose agar (PDA) plate at − 1 °C. We hence examined whether *P. glacialis* undergoes cell growth on frozen PDA at − 1 °C but found that it did not grow. This result agrees with a previous result^17^, in which *P. glacialis* could not grow at − 1 °C even on an unfrozen PDA.

Another possible physiological role of PsgIBP is to decrease the ice crystal size to prevent physical damage to the cells, as reported for other microorganisms^8^. Our experiments showed that both PsgIBP_S and PsgIBP_L isoforms function as effective IR inhibitors even at nano- to micromolar concentrations (Fig. 6B). To date, all known fungus-derived IBPs except TisIBP isoform 8 exhibit moderate TH and effective IRI activities^14,35,36^. Hence, the combination of moderate TH and high IRI activities may have significance for the survival of cold-adapted fungi in ice-laden environments.

## Conclusion

We isolated and characterized two new IBP isoforms, PsgIBP_S and PsgIBP_L, from the Arctic glacier fungus *Psychromyces glacialis*. The molecular weight typical for microbial IBPs was evaluated at 25 kDa for PsgIBP_S and at 28 kDa for PsgIBP_L, in which dimerization was highly speculated for the latter. Their TH activities were less than 1 °C at millimolar concentrations, indicating that PsgIBPs are not categorized into the hyperactive species but rather the moderately active species of IBP. Significantly, both PsgIBP isoforms can bind to the whole crystal surface of a single ice crystal, although the moderately IBPs generally bind to only specific surfaces. The two isoforms further exhibited significantly high IRI activity. These results suggest that not only the ability of whole ice-plane binding but also some factors, such as ice-binding strength, are necessary for the hyperactivity of an IBP species.

## Materials and method

### Fungal cells

*Psychromyces glacialis* (*Rhodotorula svalbardensis* strain MLB-I) was isolated from cryoconite holes in Svalbard as described previously^17^ and were cultivated in PDB medium at 4 °C.

### Expression and purification of the PsgIBP isoforms

*Psychromyces glacialis* was grown in 500 ml of potato dextrose broth (PDB) medium at 10 °C. Then, IBP expression was induced by decreasing the temperature to 4 °C. After 6–12 months, the cells were removed by centrifugation and filtration. The culture supernatant was dialyzed against pure water and subjected to our modified ice-affinity purification method (Fig. 1). Approximately 500 ml of the dialyzed solution was put into a polystyrene-foam thermal-insulation vessel and was placed in a − 3 °C-refrigerator. After a thin ice layer formed on the top surface of the sample solution, stirring was performed to prevent solutes from accumulating at the ice-solution interface. This thin ice layer was grown at − 2 °C for 10–16 h until 80% of the sample was frozen. This ice fraction was detached from the vessel, washed with cold water and melted at room temperature. The pH of the melted ice fraction was adjusted by adding 1.0 M Tris–HCl buffer (pH 8.0) up to a final concentration of 20 mM. The fraction was then loaded onto an anion-exchange High-Q column (Bio-Rad, Hercules, CA, USA) equilibrated with 20 mM Tris–HCl buffer (pH 8.0). The bound proteins were eluted with a NaCl gradient (0–150 mM). The peaks showing ice-shaping activity were independently collected, concentrated into a 5-ml volume and loaded into a Superdex200 column (GE Healthcare, Chicago, IL, USA) equilibrated with 20 mM Tris–HCl (pH 8.0) + 500 mM NaCl. The active peaks obtained with gel-filtration chromatography were collected and dialyzed against pure water. All purification procedures except for the ice-affinity purification were performed at 4 °C.

### Molecular weight estimation of the PsgIBPs with size exclusion chromatography

The molecular weight was estimated for the PsgIBP_S and PsgIBP_L samples by size-exclusion chromatography using a Superose12 column (GE Healthcare, Chicago, Illinois, USA). This column was equilibrated with 20 mM Tris–HCl (pH 8.0) + 500 mM NaCl at a flow rate of 0.8 ml/min at 4 °C. A 0.5-mL sample containing 1 mg/ml protein was loaded onto the column. The protein samples were bovine serum albumin (BSA; 66 kDa) from Sigma–Aldrich (St. Louis, MO, USA), green fluorescence protein-labeled notched-fin-eelpout-derived AFP (GFP-Nfe; 34 kDa)^37^, *Antarctomyces psychrotrophicus*-derived IBP (AnpIBP; 22 kDa)^22^, longsnout poacher-derived IBP (lpAFP; 14 kDa)^38^, and barfin plaice-derived AFP (BpAFP; 3.5 kDa)^39^. Bovine thyroglobulin (660 kDa) from Sigma–Aldrich was used to measure the void volume of the Superose12 column. A calibration line was drawn on the basis of the partition coefficients (*K*) versus the logarithm of the molecular weight of the proteins^14^. The coefficient *K* was calculated with the following equation:

$$ K = \, \left( {V_{e} {-}V_{o} } \right)/\left( {V_{t} {-}V_{o} } \right); $$

where *V*_*e*_ is the peak elution volume, *V*_*o*_ is the void volume, and *V*_*t*_ is the total bed volume of the column.

### Measurement of the thermal hysteresis activity

The thermal hysteresis activity was measured as previously described^40^. Briefly, 1 μl of the sample solution in a glass capillary was inserted into a copper holder and placed on a photomicroscope system (Leica DMLB100) equipped with a temperature controller (Linkam 10002L). The solution was flash-frozen by decreasing the temperature to − 25 °C at a rate of 55 °C/min and was then warmed up to approximately 0 °C to form a single ice crystal. At that time, the melting point (*T*_*m*_) of the sample was recorded. After the formation of a single ice crystal, the sample was incubated at *T*_*m*_ − 0.05 °C for 3 min. Then, the temperature was decreased at a rate of 0.1 °C/min. The temperature at which the ice crystal grew was recorded as the freezing point (*T*_*f*_) of the sample, which enables the calculation of TH (TH =|*T*_*m*_−*T*_*f*_ |).

### Fluorescence-based ice plane affinity (FIPA) analysis.

FIPA analysis was performed as previously described^13^. Briefly, a single ice crystal hemisphere (φ = 3 cm) attached to a “cold finger” was immersed and grown in 35 ml of fluorescently labeled IBP solution at a concentration of 25 μg/ml. The temperature of the cold-finger was initially set at − 8 °C and was gradually decreased up to − 12 °C. After growing the hemisphere to φ = 8 cm, it was illuminated under UV light and photographed in a cold room at − 1 °C. 6-(Tetramethylrhodamine-5-(and-6)-carboxamido) hexanoic acid, succinimidyl ester (5(6)-TAMRA-X SE) and rhodamine red-X, succinimidyl ester 5-isomer (Thermo Fisher Scientific, Waltham, MA, USA), both of which react with lysine residues, were used for the fluorescent labeling of RsIBPs and AnpIBPs, respectively.

### Evaluation of ice-recrystallization inhibition activity

IRI activity was measured as previously described^22^ with slight modifications. In short, morphological changes in the ice crystals that were in 30% sucrose with the presence of IBP were observed under a microscope equipped with a temperature controller (which was the same as that used in the TH measurement). Then, we can compare the size of ice crystals before and after 60 min of incubation at − 6 °C. The average size of the ice crystals was measured with ImageJ software. The concentration threshold was defined as the protein concentration at which IR did not occur. Each measurement was performed in triplicate.

## Supplementary Information

Supplementary Figures.

## Data Availability

The datasets used and/or analyzed during the current study available from the corresponding author on reasonable request.

## Abbreviations

IBP: Ice-binding protein
AFP: Antifreeze protein
TH: Thermal hysteresis
FIPA: Fluorescent-based ice plane affinity
IAP: Ice-affinity purification
MW: Molecular weight
SEC: Size-exclusion chromatography
IR: Ice-recrystallization
IRI: Ice-recrystallization inhibition
IBS: Ice-binding site
PDA: Potato dextrose agar
PDB: Potato dextrose broth
SDS-PAGE: Sodium dodecyl sulfate polyacrylamide gel electrophoresis
CBB: Coomassie Brilliant Blue
