# Supplementary material for: Adsorption of ice-binding proteins onto whole ice crystal surfaces does not necessarily confer a high thermal hysteresis activity
Source: Sci Rep. 2022 Sep 14;12:15443. doi: 10.1038/s41598-022-19803-3 (PMC9474881; doi:10.1038/s41598-022-19803-3)
Supplement: Supplementary file 1 — Supplementary Figures. [file 41598_2022_19803_MOESM1_ESM.pdf]

Supporting Information for

Adsorption of fungal ice-binding proteins to whole ice crystal surface is deficient for high thermal hysteresis activity but strongly inhibits ice recrystallization.

Tatsuya Arai§, Akari Yamauchi, Yue Yang, Shiv Mohan Singh, Yuji C. Sasaki, and Sakae Tsuda.

§Correspondence
E-mail: t.arai@edu.k.u-tokyo.ac.jp
Telephone: +81-4-7136-3892
Address: Department of Advanced Materials Science, Graduate School of Frontier Sciences, the University of Tokyo, 7H8 #609 Kiban Bldg., 5-1-5 Kashiwanoha, Kashiwa, Chiba 277-8561, Japan

This file includes:
Figure. S1, S2

Figure S1

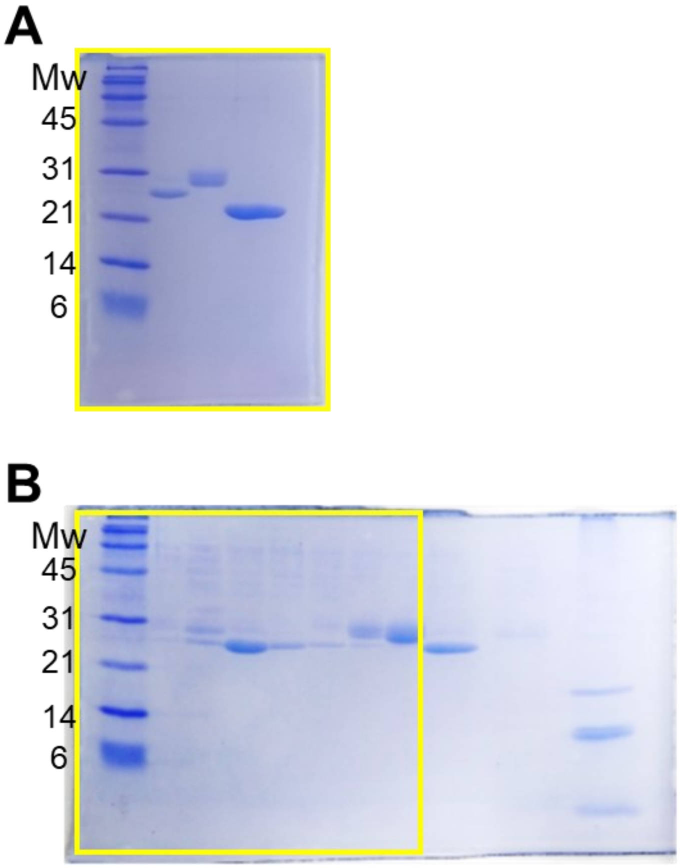

Raw uncropped images of SDS-PAGE gels for Fig. 2B (A) and Fig. 2C (B). Yellow box indicates the region used for the figure in the main text.

Figure S2

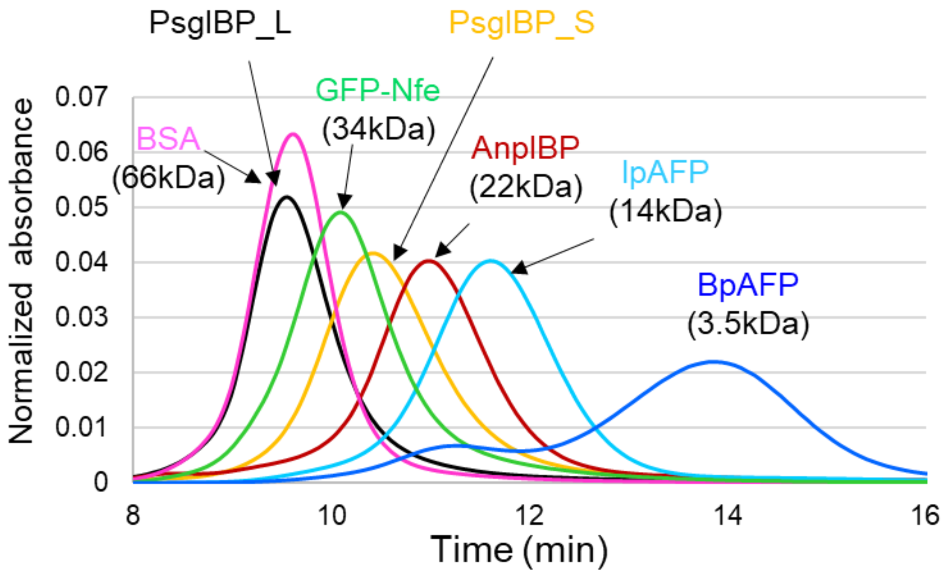

The elution profiles of the PsgIBP isoforms and those with five reference proteins obtained by using Superose12 gel chromatography. BSA: bovine serum albumin (66 kDa), GFP-Nfe: green-fluorescence protein-labeled notched fin eelpout-derived IBP (34 kDa), AnpIBP: Antarctomyces psychrotrophicus-derived IBP (22 kDa), lpAFP: Longsnout poacher-derived IBP (14 kDa), BpAFP: Barfin plaice-derived IBP (3.5 kDa).
